# Supplementary material for: APC Loss Prevents Doxorubicin-Induced Cell Death by Increasing Drug Efflux and a Chemoresistant Cell Population in Breast Cancer
Source: Int J Mol Sci. 2023 Apr 21;24(8):7621. doi: 10.3390/ijms24087621 (PMC10145529; doi:10.3390/ijms24087621)
Supplement: Supplementary file 1 [file ijms-24-07621-s001.zip › ijms-2298826-supplementary.pdf]

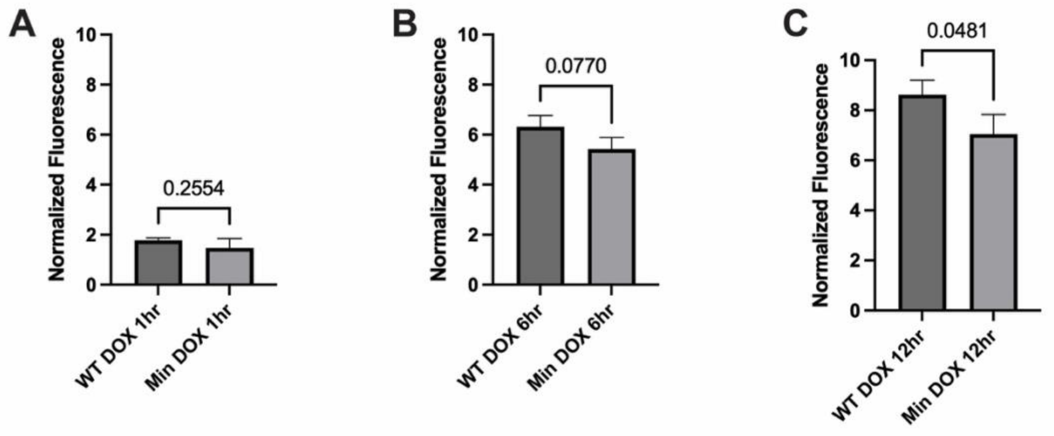

Supplemental Figure 1: DOX intracellular concentration time course. Normalized DOX fluorescence to control treated of respective genotype after 1, 6, and 12 hours of DOX treatment. Intracellular DOX concentration is significantly reduced in MMTV-PyMT;*Apc*$^{Min/+}$ cells starting at 12 hours compared to MMTV-PyMT;*Apc*$^{+/+}$ cells.

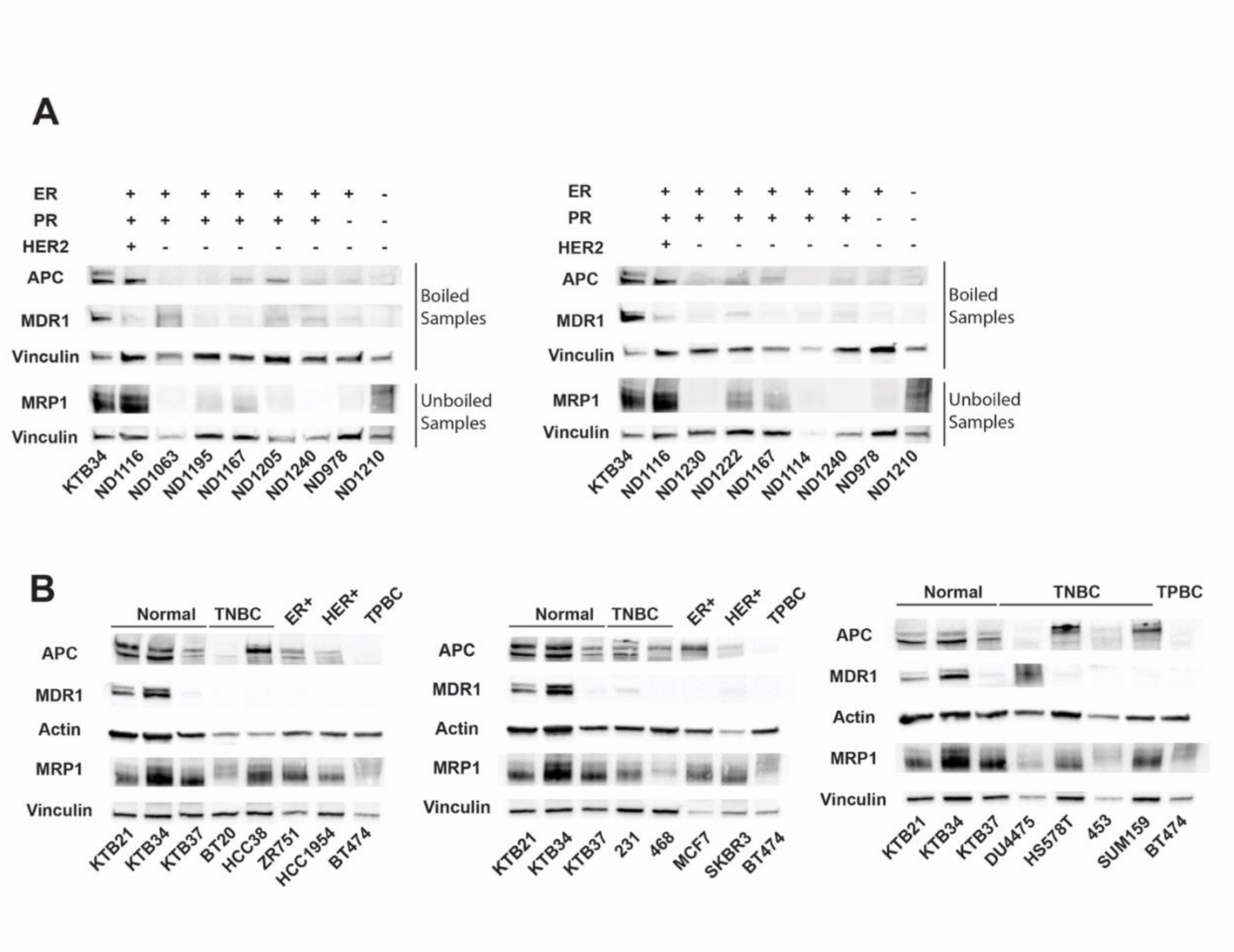

Supplemental Figure 2: Protein levels of APC, MDR1 and MRP1 for correlation studies. A) Representative westerns for APC and MDR1 for human patient tumor lysates. KTB34 is a normal breast epithelial cell line used to normalize the expression. Vinculin was used as a loading control. **B)** Representative westerns for APC and MRP1 expression in patient tumor samples. KTB34 was used to normalize, and Vinculin is the loading control. **C)** Western blot representative image for APC, MDR1, Actin, MRP1, and Vinculin in a panel of human breast cancer cell line. The average of the three normal breast epithelial cell lines (KTB21, KTB34, and KTB37) was used to normalize the expression. Actin was used as a loading control for APC and MDR1 while Vinculin was the loading control for MRP1. TNBC= triple negative breast cancer (ER-, PR- and Her2-); TPBC= triple positive breast cancer (ER+, PR+ and Her2+).

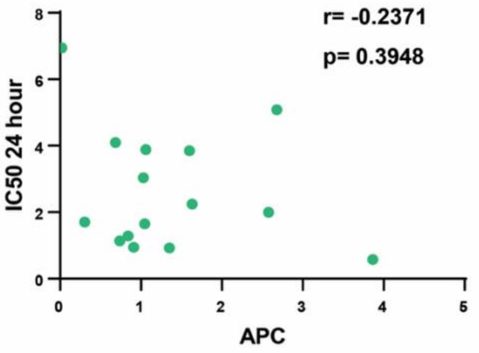

Supplemental Figure 3: DOX IC50s trends with low APC levels at 24 hours. A) Using a panel of human breast cancer cell lines, the expression of APC was correlated to DOX IC50s. Protein level and DOX IC50 concentrations were normalized to the average of the normal breast epithelial cell lines. Decreasing APC protein level trends with increased DOX IC50s at 24 hours (Pearson's r= -0.2371) but this trend is not statistically significant (p= 0.3948).

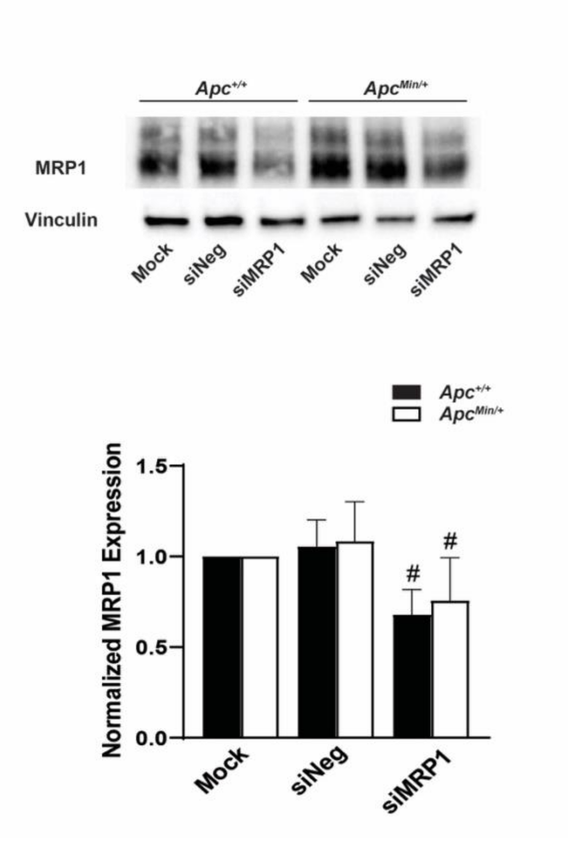

Supplemental Figure 4: siMRP1 knockdown validation. A) Representative western blots and quantification shows that siMRP1 transfection decreased MRP1 expression in both MMTV-PyMT;*Apc*$^{+/+}$ cells and MMTV-PyMT;*Apc*$^{Min/+}$ cells. #P < 0.05 comparing MMTV-PyMT;*Apc*$^{+/+}$ cells and MMTV-PyMT;*Apc*$^{Min/+}$ cells with or without treatment to control-treated MMTV-PyMT;*Apc*$^{+/+}$ cells.

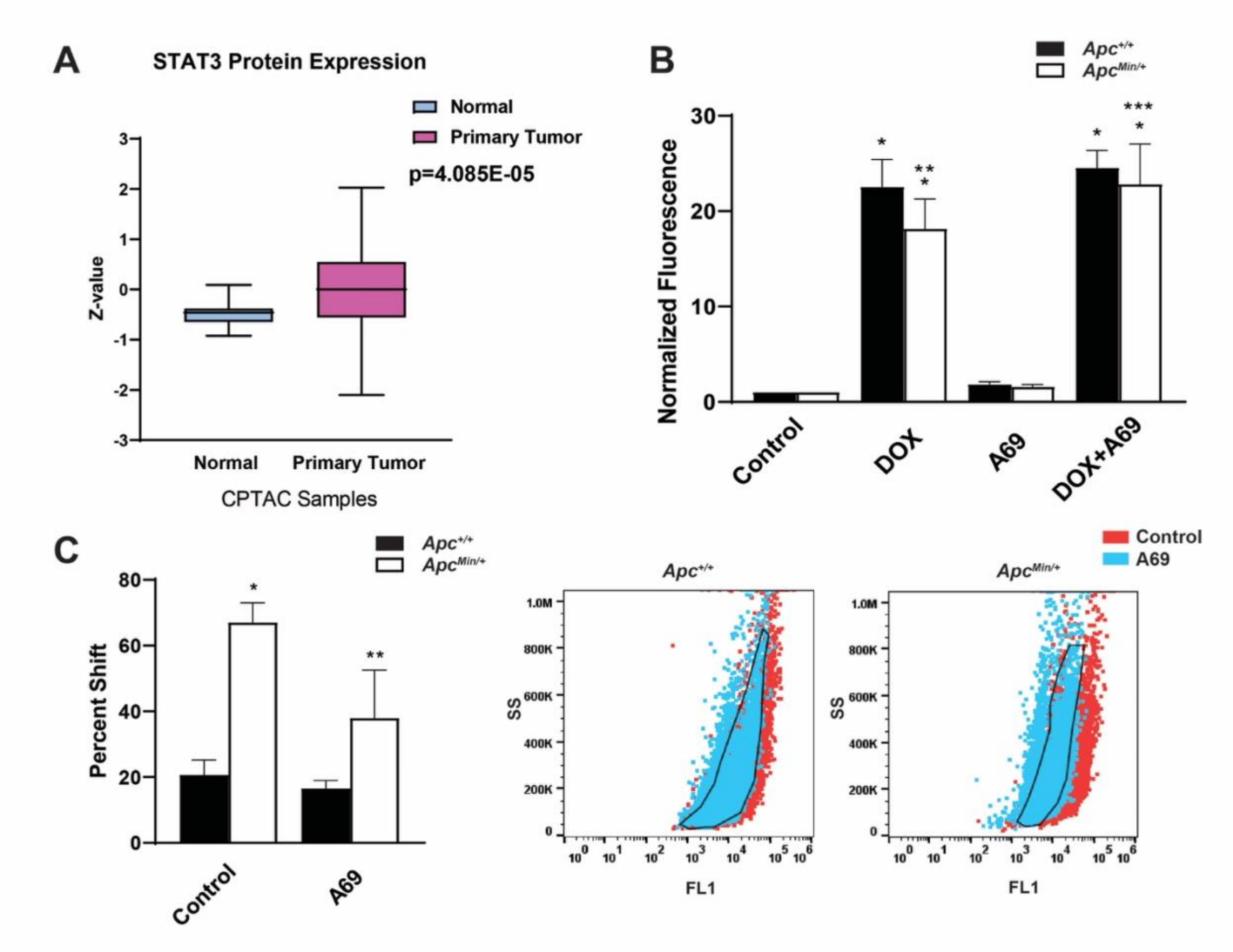

Supplemental Figure 5: STAT3 activation in breast cancer regulates DOX efflux and TIC population. A) CPTAC samples show increased STAT3 in primary tumors compared to normal breast tissue. **B)** Combination therapy with a STAT3 inhibitor, A69, increased DOX intracellular accumulation after 48 hours of treatment in MMTV-PyMT;*Apc*$^{Min/+}$ cells compared to DOX treatment only. Combination treatment did not augment DOX accumulation in MMTV-PyMT;*Apc*$^{+/+}$ cells. **C)** A69 treatment decreased the ALDH^{High} population in MMTV-PyMT;*Apc*$^{Min/+}$ cells as compared to control, but did not bring the ALDH^{High} cells to the level of MMTV-PyMT;*Apc*$^{+/+}$ cells which could be due to other factors supporting the ALDH^{High} cell population in MMTV-PyMT;*Apc*$^{Min/+}$ cells. Representative population shifts demonstrate the change between control and A69 treatment compared to the black outline of untreated control. *P < 0.05 comparing treated cells to control-treated cells or control-treated MMTV-PyMT;*Apc*$^{Min/+}$ cells to control-treated MMTV-PyMT;*Apc*$^{+/+}$ cells. **P < 0.05 as compared with DOX-treated MMTV-PyMT;*Apc*$^{+/+}$ cells or A69 treated MMTV-PyMT;*Apc*$^{Min/+}$ cells to control treated MMTV-PyMT;*Apc*$^{Min/+}$ cells ***P < 0.05 comparing combination treatment in MMTV-PyMT;*Apc*$^{Min/+}$ cells to single treatment.

Cell Line	APC Expression	IC_{50} (uM)at 24hr
KTB21	1.35	12.9
KTB34	0.91	13.2
KTB37	0.74	15.8
MCF7	1.63	31.3
ZR751	1.60	53.8
HCC1954	1.06	54.3
SKBR3	1.05	23.0
BT474	0.02	96.9
BT20	0.68	57.2
HCC38	3.87	8.0
HS578T	2.58	27.8
MDA-MB-231	1.03	42.4
MDA-MB-453	0.84	18.0
MDA-MB-468	0.30	23.8
SUM159	2.68	71.0

Supplemental Table 1. IC50 correlation with APC expression in human breast epithelial or breast cancer cell lines.
